# CHILDREN CAREGIVERS’ EXPERIENCES OF OLDER ADULTS WITH DEMENTIA IN CARE TRANSITION: A QUALITATIVE SYSTEMATIC REVIEW

**DOI:** 10.1093/geroni/igz038.3098

**Published:** 2019-11-08

**Authors:** Rungnapha Khiewchaum

**Affiliations:** Sinclair School of Nursing, University of Missouri, Columbia, Columbia, Missouri, United States

## Abstract

Background/Purpose: Young and adult children have experienced caring for people who have been diagnosed with dementia. Caregiving needs affect family members who become the primary caregivers in care transition from hospital to home. This study aims to synthesize primary qualitative research on the experiences of children caregivers of older adults with dementia. Method: This is a systematic review describing young and adult child caregivers’ experiences in caring for patient with dementia in home-based care. Data sources were published literature written in English from CINAHL, Scopus, PubMed, and PsychoINFO (published from January 1976 to October 2018). The thematic synthesis approach was also applied to generate theory generating meta-synthesis research (TGMS). and to describe the process of caring for demented patients by caregivers. Result: Eight primary studies reporting 388 potential studies were included. Four themes emerged: 1) well-being which included encouraging and destructive well-being; 2) role transition which included positive or negative role transformations; 3) caregiver needs which included medical and nursing information or knowledges and health care services/community services; and 4) the challenge of dementia which included symptoms of dementia which were impairing. Conclusion The findings of this meta-synthesis study support evidence of well-being among adult children in caring for people with dementia in transition phases. We present thematic synthesis that could be useful to professionals working with caregivers and patients with dementia. We suggest that research importance should shift towards the development and evaluation of care transitions intervention, especially professionals preparing support after diagnosis.

